# Imaging findings of primary hepatic carcinoid tumor with an emphasis on MR imaging: case study

**DOI:** 10.1186/2193-1801-3-607

**Published:** 2014-10-17

**Authors:** Makoto Ichiki, Norifumi Nishida, Akira Furukawa, Shuzo Kanasaki, Shinichi Ohta, Yukio Miki

**Affiliations:** Department of Radiology, Osaka City General Hospital, 2-13-22 Miyakojima-hondori, Miyakojima-ku, Osaka, Japan; Department of Radiology, Osaka City University Graduate School of Medicine, 1-4-3, Asahi-machi, Abeno-ku, Osaka, Japan; Department of Radiological Sciences, Tokyo Metropolitan University, 1-1 Minami-Osawa, Hachioji-shi, Tokyo, Japan; Department of Radiology, Koseikai Takeda Hospital, 841-5 Higashishiokoji-cho, Shiokojidori Nishinotoin Higashi-iru, Shimogyo-ku, Kyoto, Japan; Department of Radiology, Shiga University of Medical Science, Seta Tsukinowa-cho, Otsu, Shiga, Japan

**Keywords:** Carcinoid, Liver, Magnetic resonance imaging

## Abstract

Carcinoid tumors are slow-growing tumors originating in the neuroendocrine cells, and occur most frequently within the gastrointestinal tract. Although the liver is the most common site for metastatic carcinoid tumors, primary hepatic carcinoid tumors are exceedingly rare and reports of the imaging findings have been very scarce. We herein report imaging findings with an emphasis on magnetic resonance imaging in two cases of primary hepatic carcinoid tumors. In both cases, the tumors showed cystic areas with hemorrhagic components and early enhanced solid areas.

## Background

Carcinoid tumors are slow-growing tumors originating in the neuroendocrine cells, and occur most frequently within the gastrointestinal tract (Maggard et al. 2004; Nikfarjam et al. 2004). Although they preferentially metastasize to the liver, primary hepatic carcinoid tumors are exceedingly rare (Gravante et al. 2008; Nikfarjam et al. 2004) and there are insufficient detailed data on these tumors in the literature. In addition, most previous reports of this tumor have concerned histological or surgical findings, and reports of the imaging findings have been very scarce.

We herein present two cases of primary hepatic carcinoid tumors and describe the characteristic of imaging findings, with an emphasis on magnetic resonance (MR) imaging. To the best of our knowledge, there have been no report describing the MR imaging findings with the hepatocyte-specific contrast media like one of our cases.

## Case descriptions

### Case 1

**Figure 1:**
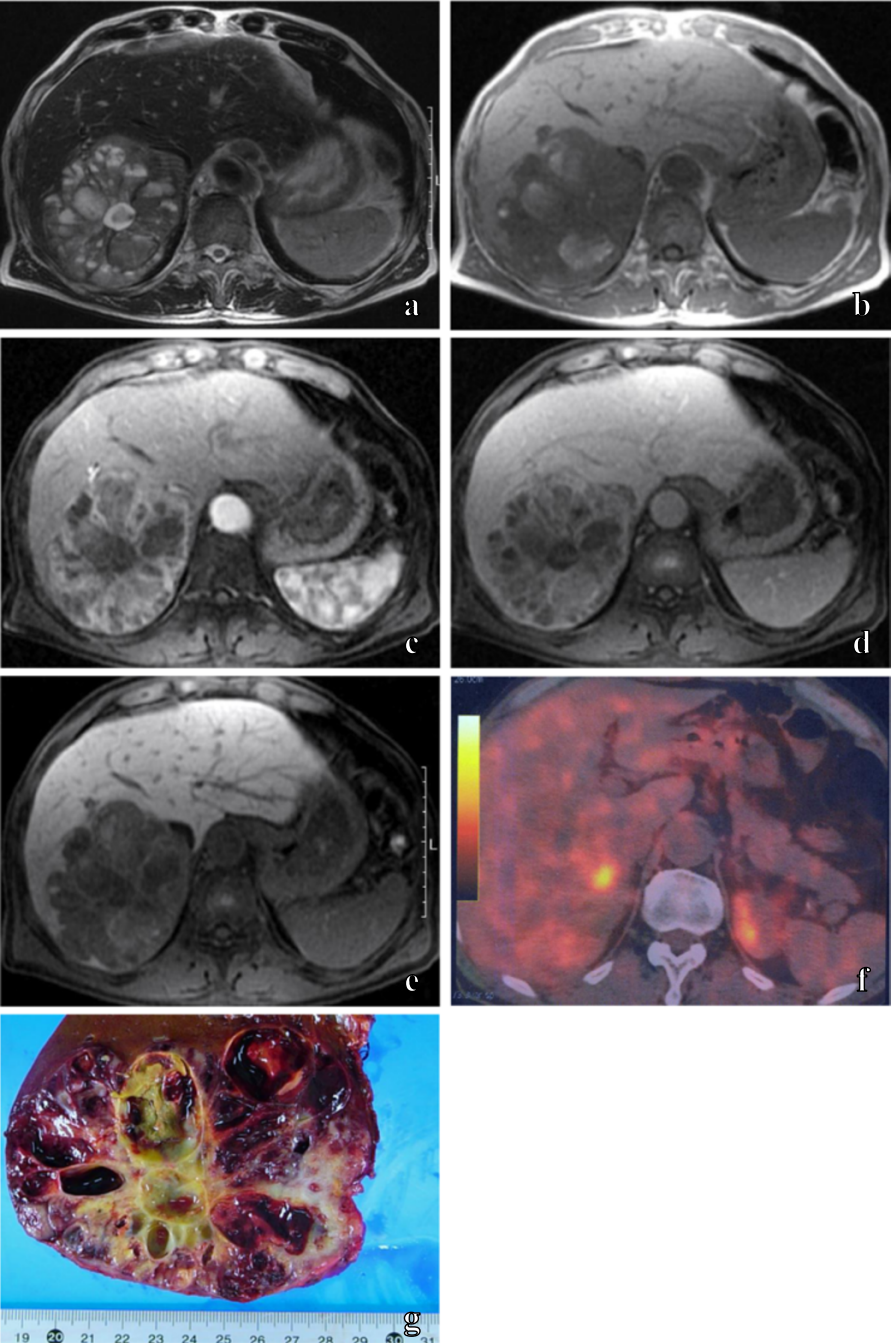
**Primary hepatic carcinoid tumor in 70-year-old male.** An axial T2-weighted MR image **(a)** shows the hepatic mass, which included multiple cystic areas with shading. An axial T1-weighted MR image **(b)** shows some hyperintense cystic areas in the hepatic mass. Axial fat-suppressed dynamic MR images with Gd-EOB-DTPA show the prolonged enhancement of solid areas and the non-enhancing multiple cystic areas of the hepatic mass from the early phase **(c)** to the late phase **(d)**. An axial fat-suppressed MR image with Gd-EOB-DTPA in the hepatobiliary phase **(e)** shows the hypointense hepatic mass, the extent of which is clearly delineated by increasing the signal intensity of the normal liver parenchyma. 18 F-FDG PET/CT **(f)** shows no abnormal uptake of FDG in most of the hepatic mass, and increased uptake of FDG in the fraction of the hepatic mass. The cut surface of the resected specimen **(g)** shows a hepatic mass with multiple hemorrhagic cystic areas.

A 70-year-old male with past medical history of a squamous cell carcinoma of the skin underwent a computed tomography (CT) examination for the assessment of distant metastasis. He was asymptomatic. Unenhanced CT showed a heterogeneous hypodense mass mainly in the posterior segment of the right hepatic lobe. On enhanced CT, multiple cystic areas without contrast enhancement became clear by the enhancing solid areas in the hepatic mass. His laboratory data showed high levels of serum γ-glutamyl transpeptidase (75 IU/L) and leucine aminopeptidase (136 IU/L). Tumor marker assays showed high levels of serum carcinoembryonic antigen (5.7 ng/mL) and carbohydrate antigen 19-9 (154 U/mL). Abdominal ultrasound showed the mainly hyperechoic mass with multiple cystic areas. Abdominal MR imaging showed the hepatic mass with solid and multiple cystic areas. T2-weighted MR images showed multiple cystic areas with shading in the hepatic mass (Figure 1a). T1-weighted MR images showed some hyperintense cystic areas in the hepatic mass (Figure 1b). These findings suggested a hemorrhagic component in the cysts. On dynamic MR images with gadolinium ethoxybenzyl diethylenetriamine pentaacetic acid (Gd-EOB-DTPA), strong enhancement of solid areas in the hepatic mass was prolonged from the early phase to the late phase (Figure 1c, d). MR images with Gd-EOB-DTPA in the hepatobiliary phase showed the hypointense hepatic mass, the extent of which was clearly delineated by increasing the signal intensity of the normal liver parenchyma (Figure 1e). 18 F-fluorodeoxyglucose (FDG) positron emission tomography (PET)/CT showed no abnormal uptake of FDG in most of the hepatic mass, and an increased uptake of FDG in the fraction of the hepatic mass (Figure 1f). An extended right lobectomy was performed. Macroscopic inspection of the abdominal cavity during the operation demonstrated no other abnormal findings. The cut surface of the resected specimen showed a hepatic mass measuring 11 × 8 cm with multiple hemorrhagic cystic areas (Figure 1g). Microscopically, the tumor was composed of columnar cells arranged in trabecular and ribbon patterns. Immunohistochemically, the tumor cells were positive for synaptophysin and CD56 and negative for chromogranin A. Only a few tumor cells were positive Ki-67. So a diagnosis of carcinoid tumor was made. No extrahepatic lesions were found by upper endoscopy, colonoscopy, small bowel series or chest CT. Recurrences were found in the residual liver five months after the operation, and transcatheter arterial chemoembolization was performed for the lesions. The patient was doing well at the most recent follow-up examination, performed 22 months after the operation.

### Case 2

**Figure 2:**
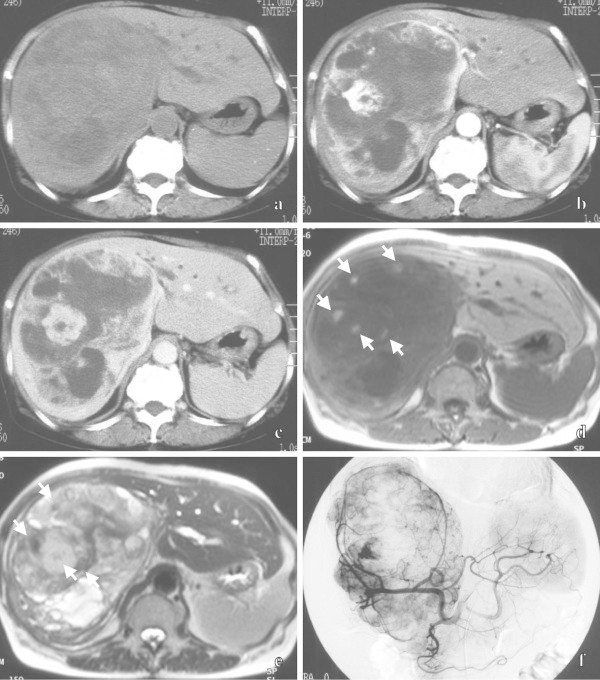
**Primary hepatic carcinoid tumor in 74-year-old female.** Unenhanced CT **(a)** shows a heterogeneous hypodense mass mainly in the right lobe of the liver. Dynamic CT shows prolonged, enhancing, irregular solid areas and non-enhancing areas of the hepatic mass from the early phase **(b)** to the late phase **(c)**. An axial T1-weighted MR image **(d)** shows the mainly hypointense mass, including scattered hyperintense cystic areas (arrows). An axial T2-weighted MR image **(e)** shows the mainly hyperintense mass, including cystic areas with shading (arrows). A celiac arteriogram in the early phase **(f)** shows an irregular tumor stain, which is supplied by the stretching right and middle hepatic arteries.

A 74-old-year female had experienced abdominal distension for three years. She was hospitalized for a detailed examination and medical treatment, because her abdominal pain had gradually intensified. Her past medical history included bronchial asthma, but she had not experienced any asthma attacks after she was in 30s. Her laboratory data showed a high level of serum γ-glutamyl transpeptidase (64 IU/L). Abdominal unenhanced CT showed a heterogeneous hypodense mass 12 cm in diameter, mainly in the right lobe of the liver (Figure 2a). On dynamic CT, irregular solid areas with strong enhancement, which was prolonged from the early phase to the late phase, was observed with non-enhancing areas mixed in the hepatic mass (Figure 2b, c). The hepatic mass was mainly hypointense on T1-weighted MR images (Figure 2d) and hyperintense on T2-weighted MR images (Figure 2e). Cystic areas which were hyperintense on T1-weighted MR images and showed shading on T2-weighted MR images were scattered in the mass, thus suggesting a hemorrhagic component in the cysts. Abdominal arteriograms showed irregular tumor staining, which was supplied by the stretching right and middle hepatic arteries (Figure 2f). Based on these imaging findings, hepatic hemangioma with remarkable degeneration was suspected. Transcatheter arterial embolization was performed for the hepatic mass, and the size of mass was reduced to 10 cm in diameter. After a follow-up of 43 months, an extended right lobectomy was performed because the mass had increased in size, and also due to the fact that her abdominal pain had intensified. A macroscopic inspection of the abdominal cavity during the operation demonstrated no other abnormal findings. The cut surface of the resected specimen showed a hepatic mass with multiple hemorrhagic cystic areas. Microscopically, the tumor was composed of homogeneous round cells arranged in trabecular and nesting patterns. Some of the cells formed rosette structures. Histopathologically, the diagnosis was a carcinoid tumor. No extrahepatic lesions were found by upper endoscopy, colonoscopy or chest X-rays. No recurrence was found at 30 months after the operation.

## Discussion and evaluation

Although the liver is the most common site for metastatic carcinoid tumors, primary hepatic carcinoid tumors are exceedingly rare (Gravante et al. 2008; Nikfarjam et al. 2004), and their identity must be demonstrated by confirmation that there is no carcinoid in other sites at the time of the operation and by various imaging examinations. Primary hepatic carcinoid tumors had some common clinical characteristics (Gravante et al. 2008; Iwao et al. 2001; Lin et al. 2009; Fenoglio et al. 2009; Nikfarjam et al. 2004). They frequently occurred in middle age and were detected slightly more often in females than males. A relatively large number of patients presented with abdominal pain or no symptoms, whereas a few patients presented with carcinoid syndrome. Therefore, most such tumors were incidentally found as giant masses in spite of their slow-glowing nature.

Although few previous reports have shown the characteristics of imaging findings of primary hepatic carcinoid tumors owing to their rarity, it has been thought, based on previous reports and our present cases, that the relatively characteristic imaging findings include cystic areas with hemorrhagic components (Takayasu et al. 1992; Abdel Wahab et al. 2006; Komatsuda et al. 2005; Ulusan et al. 2005; Touloumis et al. 2008; Imaoka et al. 1993; Fujino et al. 1998; Oh et al. 1998; Kehagias et al. 1999; Hirata et al. 2002; Aoki et al. 1992; Yeung et al. 2008; Shih et al. 2005) and early enhanced solid areas (Iwao et al. 2001; Lin et al. 2009; Takayasu et al. 1992; Abdel Wahab et al. 2006; Komatsuda et al. 2005; Ulusan et al. 2005; Touloumis et al. 2008; Iimuro et al. 2002; Gao J et al. 2011; Shah et al. 2007).

Some cystic areas of the tumors in our present cases showed hyperintensity on T1-weighted MR images and shading on T2-weighted MR images, and were confirmed to include hemorrhagic components in the resected specimens. In several previous reports, cystic areas of primary hepatic carcinoid tumors were also confirmed as vascular lakes, which included hemorrhagic components histopathologically (Takayasu et al. 1992; Abdel Wahab et al. 2006; Imaoka et al. 1993; Fujino et al. 1998; Oh et al. 1998; Kehagias et al. 1999; Hirata et al. 2002; Aoki et al. 1992) and would have originated in frequent intratumoral bleeding. Table 1 shows the reported MR imaging findings of primary hepatic carcinoid tumors. Several previous reports, as well as our present cases, showed primary hepatic carcinoid tumors with hyperintense areas on T1-weighted MR images, and some of them were identified to have hemorrhagic components in the resected specimens (Takayasu et al. 1992; Imaoka et al. 1993). In contrast, a few reports identified histopathologically hemorrhagic components at hyperintense areas on T2-weighted MR images (Takayasu et al. 1992; Abdel Wahab et al. 2006; Fujino et al. 1998). It is thought that cystic areas of primary hepatic carcinoid tumors can show various signal patterns depending on the amount and duration of bleeding.

**Table 1:** **Summary of the reported MR imaging findings of primary hepatic carcinoid tumors**

Author (year)	Age/sex	T1-weighted imaging findings	T2-weighted imaging findings
Iwao et al. (2001)	75/M	Hypointense masses	Hyperintense masses
Fenoglio et al. (2009)	65/M	A hypointense mass	A weak irregular hyperintense mass
Takayasu et al. (1992)	30/F	A hypointense mass with multiple small hyperintense areas	A hyperintense mass with small more hyperintense and hypointense areas
	74/F	A hypointense mass with hyperintense foci	A hyperintense mass with more hyperintense zonal areas
Abdel Wahab et al. (2006)	62/F	A heterogeneous hypointense mass	A mass with hyperintense areas of cystic degeneration
	55/F	NA	A hyperintense mass with areas of intermediate signal intensity
	52/F	NA	A hyperintense mass with an area of intermediate signal intensity
Imaoka et al. (1993)	56/F	A heterogeneous hypointense mass with a small hyperintense spot	A hyperintense mass containing hypointense septae
Fujino et al. (1998)	40/F	A heterogeneous hypointense area	A hyperintense mass containing more hyperintense areas
Kehagias et al. (1999)	57/M	A mainly isointense mass relative to the normal liver with several hypointense areas	A mass composed of central and multiple peripheral hyperintense areas
Yeung et al. (2008)	35/F	Two hepatic masses with thick hypointense walls and hyperintense centers	NA
Iimuro et al. (2002)	71/M	A hypointense mass	A heterogeneous hyperintense mass
Our cases	70/M	A mass including some hyperintense cystic areas	A mass including multiple cystic areas with shading
	74/F	A mainly hypointense mass including scattered hyperintense cystic areas	A mainly hyperintense mass including cystic areas with shading

F: female, M: male, NA: not available.

Table 2 shows the results of the reported dynamic studies of primary hepatic carcinoid tumors. The solid areas were densely stained in the early phase of dynamic studies. In the late phase, some cases had less enhanced staining to the surrounding liver parenchyma (Iwao et al. 2001; Lin et al. 2009; Takayasu et al. 1992; Abdel Wahab et al. 2006; Komatsuda et al. 2005 Iimuro et al. 2002; Gao et al; 2011) and the others showed a prolonged and strengthened enhancement (Takayasu et al. 1992; Abdel Wahab et al. 2006; Ulusan et al. 2005; Touloumis et al. 2008). Our present cases showed prolonged enhancements from the early phases to the late phases of dynamic studies. Irregular, prolonged and strengthened enhancements of primary hepatic carcinoid tumors in the late phase of dynamic studies appear to originate in fibrous tissues (Ulusan et al. 2005). MR imaging with Gd-EOB-DTPA for Case 1 not only evaluated the hemodynamics of the tumor in the dynamic study, but also clearly described the shape of the tumor without hepatocyte function owing to increasing signal intensity of the normal liver parenchyma in the hepatobiliary phase. The use of hepatocyte-specific contrast media is expected to enable a more accurate diagnosis.

**Table 2:** **Summary of the reported dynamic studies of primary hepatic carcinoid tumors**

Author (year)	Age/sex	Modality	Early phase	Late phase
Iwao et al. (2001)	75/M	CT	Peripherally enhancing masses	Hypodense masses, some of which were accompanied by a weakly enhancing central area
Lin et al. (2009)	48/F	CT	An enhancing nodule	A nodule with washout
Takayasu et al. (1992)	30/F	CT	An enhancing mass with spotty nonenhancing areas	An iso- to slight hypodense mass with more hypodense foci
	74/F	CT	NA	A mass with slight enhancement
Abdel Wahab et al. (2006)	55/F	CT	A marginally enhancing mass	A mass with complete fill-in enhancement
	52/F	CT	A marked heterogeneously enhancing mass	A mass with washout
Komatsuda et al. (2005)	82/M	US	A marked enhancing mass except for cystic areas	A less enhancing mass than the surrounding liver
Ulusan et al. (2005)	46/F	CT	A peripherally enhancing mass	A centrally enhancing mass
Touloumis et al. (2008)	65/F	MRI	Progressive enhancing lesions	
Iimuro et al. (2002)	71/M	CT	One homogeneously enhancing mass and another ringed enhancing mass	Two Masses with enhancing parts that became hypodense compared with the liver
Gao et al. (2011)	53/F	CT	An enhancing nodule	A nodule with washout
Shah et al. (2005)	46/F	MRI	An isolated arterial enhancing lesion	NA
Our cases	70/M	MRI	A mass including solid areas with strong enhancement	A mass including solid areas with prolonged enhancement and non-enhancing multiple cystic areas
	74/F	CT	A mass including irregular solid areas with strong enhancement	A mass including solid areas with prolonged enhancement and non-enhancing areas

F: female, M: male, CT: computed tomography, US: ultrasound, MRI: magnetic resonance imaging, NA: not available.

PET for Case 1 showed no abnormal uptake of FDG in most of the hepatic mass. PET is not generally useful, because carcinoid tumors are slow-growing tumors and have no hypermetabolic activity (Yeung et al. 2008).

Carcinoid tumors frequently metastasize, and the liver is the most common site. Typical imaging findings of metastatic hepatic carcinoid tumors by previous reports included solid, hypervascular and multiple lesions, whereas cystic areas were rarely observed, unlike in primary hepatic carcinoid tumors (Salamone et al. 2010). Stella et al. (2006) concluded that the cystic degeneration of a metastatic hepatic carcinoid tumor originated in necrosis over a ten-year period according to one case report. Primary hepatic carcinoid tumors can be rarely found as early solid tumors without cystic areas, because they can be found incidentally, and are less frequently associated with carcinoid syndrome than metastasis. The tumors in our two cases were both large, and thus they appear to include cystic areas due to their long period of growth.

## Conclusions

The present two cases of primary hepatic carcinoid tumor showed similar imaging findings. It has been thought, based on previous reports and our present cases, that the relatively characteristic imaging findings include cystic areas with hemorrhagic components and early enhanced solid areas. Although a primary hepatic carcinoid tumor needs to be differentiated from hepatocellular carcinoma, hemangioma, cystadenocarcinoma, sarcoma and hypervascular metastasis (such as renal cell carcinoma and islet cell tumor) in diagnostic imaging, this may be difficult owing to the rarity of this tumor. However, hepatic masses including cystic areas with hemorrhagic components and early enhanced solid areas on MR imaging should thus lead to a suspicion of a primary hepatic carcinoid tumor.

## Abbreviations

MR: Magnetic resonance
CT: Computed tomography
Gd-EOB-DTPA: Gadolinium ethoxybenzyl diethylenetriamine pentaacetic acid
FDG: Fluorodeoxyglucose
PET: Positron emission tomography.
